# Fibroblast growth factor homologous factors tune arrhythmogenic late Na_V_1.5 current in calmodulin binding–deficient channels

**DOI:** 10.1172/jci.insight.141736

**Published:** 2020-10-02

**Authors:** Jeffrey Abrams, Daniel Roybal, Nourdine Chakouri, Alexander N. Katchman, Richard Weinberg, Lin Yang, Bi-xing Chen, Sergey I. Zakharov, Jessica A. Hennessey, Uma Mahesh R. Avula, Johanna Diaz, Chaojian Wang, Elaine Y. Wan, Geoffrey S. Pitt, Manu Ben-Johny, Steven O. Marx

**Affiliations:** 1 Division of Cardiology, Department of Medicine,; 2 Department of Pharmacology, and; 3 Department of Physiology and Cellular Biophysics, Vagelos College of Physicians and Surgeons, Columbia University, New York, New York, USA.; 4 Division of Cardiology, Department of Medicine, Duke University Medical Center, Durham, North Carolina, USA.; 5 Cardiovascular Research Institute, Weill Cornell Medical College, New York, New York, USA.

**Keywords:** Cardiology, Arrhythmias, Calmodulin, Sodium channels

## Abstract

The Ca^2+^-binding protein calmodulin has emerged as a pivotal player in tuning Na^+^ channel function, although its impact in vivo remains to be resolved. Here, we identify the role of calmodulin and the Na_V_1.5 interactome in regulating late Na^+^ current in cardiomyocytes. We created transgenic mice with cardiac-specific expression of human Na_V_1.5 channels with alanine substitutions for the IQ motif (IQ/AA). The mutations rendered the channels incapable of binding calmodulin to the C-terminus. The IQ/AA transgenic mice exhibited normal ventricular repolarization without arrhythmias and an absence of increased late Na^+^ current. In comparison, transgenic mice expressing a lidocaine-resistant (F1759A) human Na_V_1.5 demonstrated increased late Na^+^ current and prolonged repolarization in cardiomyocytes, with spontaneous arrhythmias. To determine regulatory factors that prevent late Na^+^ current for the IQ/AA mutant channel, we considered fibroblast growth factor homologous factors (FHFs), which are within the Na_V_1.5 proteomic subdomain shown by proximity labeling in transgenic mice expressing Na_V_1.5 conjugated to ascorbate peroxidase. We found that FGF13 diminished late current of the IQ/AA but not F1759A mutant cardiomyocytes, suggesting that endogenous FHFs may serve to prevent late Na^+^ current in mouse cardiomyocytes. Leveraging endogenous mechanisms may furnish an alternative avenue for developing novel pharmacology that selectively blunts late Na^+^ current.

## Abstract 2

Complete protection from late Na+ current in murine cardiomyocytes expressing NaV1.5 channels devoid of apo-calmodulin binding is imparted by endogenously expressed FGF13.

## Graphical abstract

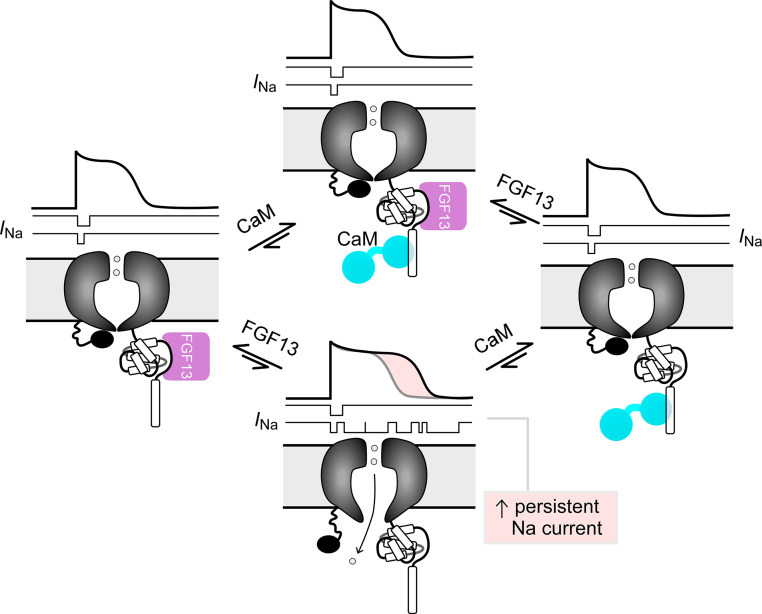

## Introduction

**Figure 1:**
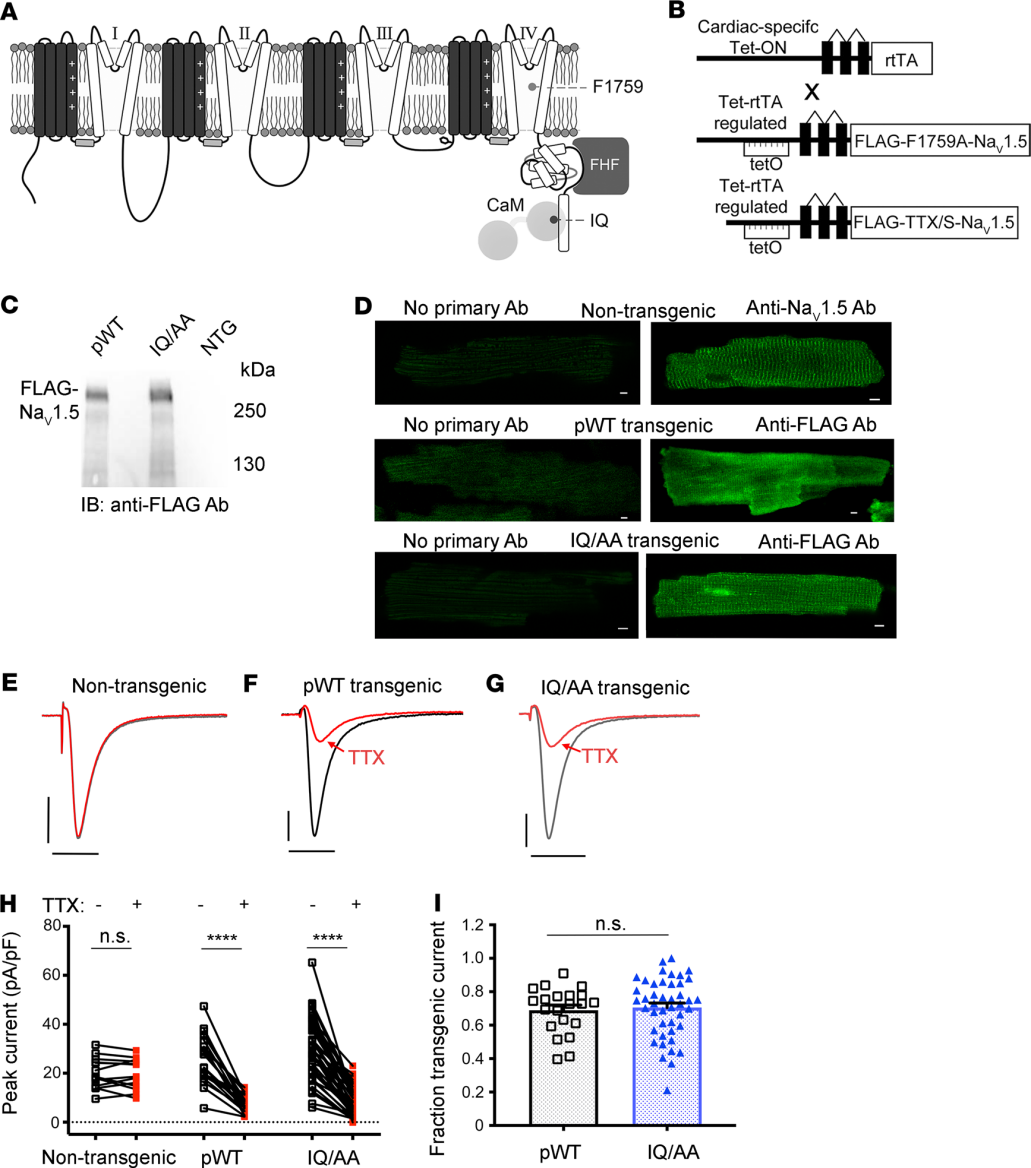
Cardiac-specific, FLAG-tagged TTX-sensitive Na_V_1.5-expressing transgenic mice. (**A**) Diagram showing Na_V_1.5. The pore-forming α-subunit is a pseudotetramer of transmembrane domains (I–IV) linked by intracellular loops. The channel’s inactivation gate is in the III–IV linker. The best-established CaM binding site is on the C-terminal domain, where the FHF binding site also resides. (**B**) Schematic of binary transgene system. The expression of reverse tetracycline-controlled transactivator (rtTA) is driven by the cardiac-specific α-myosin heavy chain promoter. The cDNAs for FLAG-F1759A-Na_V_1.5 or FLAG-tagged TTX-sensitive Na_V_1.5 were ligated behind 7 tandem *tetO* sequences. (**C**) Anti-FLAG antibody immunoblots of cleared lysates of hearts from pWT, IQ/AA, and nontransgenic mice. Representative images of 3 independent experiments. (**D**) Immunostaining of nontransgenic, pWT, and IQ/AA mice cardiomyocytes. Nontransgenic cardiomyocytes: primary antibody, anti-Na_V_1.5 antibody. pWT and IQ/AA cardiomyocytes: primary antibody, anti-FLAG antibody. FITC-conjugated secondary antibody was used for all experiments. Scale bar: 5 μm. Representative of 20 cardiomyocytes from at least 3 independent cardiomyocyte isolations for all groups. (**E–G**) Exemplar whole cell Na^+^ current trace of ventricular cardiomyocyte from nontransgenic, pWT, and IQ/AA transgenic mice in the absence (black) and presence (red) of 20 nM TTX. Representative of *n* = 13, 21, and 44 cells, from left to right. Vertical scale bars: 10 pA/pF; horizontal scale bars: 5 ms. (**H**) Graph showing effect of 20 nM TTX on peak Na^+^ current. ****P < 0.0001 by paired *t* test. For nontransgenic, *P* = 0.61. *n* = 13, 21, and 44 cells from left to right. (**I**) Graph of fraction transgenic Na^+^ current for pWT and IQ/AA. Mean ± SEM. *n* = 21 and 44 cells from left to right. *P* = 0.73 by *t* test.

Voltage-gated Na_V_ channels initiate action potentials in excitable tissue and are fundamental to defining cellular excitability. *SCN5A*-encoded Na_V_1.5 is the major cardiac Na_V_ channel, and its dysfunction is linked to multiple cardiac disorders. Voltage-gated Na^+^ channels consist of 4 transmembrane domains, each containing 6 transmembrane α-helices, connected by intracellular linkers, and intracellular N-terminal and C-terminal domains (Figure 1A). Each domain is composed of a voltage sensor (S1–S4) and pore-forming (S5 and S6) transmembrane helices that also serve as an intracellular gate. Voltage sensor movement drives channel openings, while ensuing channel inactivation depends on the allosteric blockade of ion conduction triggered by the interaction of the hydrophobic IFM motif in the III–IV linker with a hydrophobic pocket between domains III and IV (1). Impaired inactivation leads to late inward Na^+^ current that can cause cardiac arrhythmias or dilated cardiomyopathy (2, 3). Increased inward late Na^+^ influx also underlies *SCN5A*-mediated long QT syndrome type 3 (LQT3) (4). Increased late Na^+^ current is sufficient to cause structural and electrical remodeling in the atria of mice, leading to spontaneous atrial fibrillation (5). In contrast, loss-of-function mutations in Na_V_1.5 or its auxiliary subunits cause the arrhythmogenic Brugada syndrome (6). Determining how changes in Na^+^ channel structure (7) and interactome cause functional alterations in channel properties that beget cardiac disease is critical to devise new therapies.

The Ca^2+^-binding protein calmodulin (CaM) has emerged as a pivotal player in tuning cardiac Na^+^ channel function, although the impact of this regulation for cardiac function and disease remains to be fully resolved. CaM regulation of Na_V_ channels has been the subject of intense scrutiny since the recognition of a CaM binding site within the IQ motif on the Na_V_1.2 C-terminal domain 2 decades ago (8). CaM has multiple points of contact on Na_V_1.5. The C-terminal domain of Na_V_1.5 contains a canonical apoCaM “IQ” binding motif (Ile1908 and Gln1909) that the CaM C-lobe buries (9–12). Indeed, mutations of the IQ motif that disrupt CaM interaction increased late Na^+^ current in cells heterologously expressing Na_V_1.5, suggesting a role for CaM in Na_V_ inactivation (13). Human mutations within the IQ motif or in close vicinity within the CaM binding pocket — including Q1909R, E1901Q, and R1913H — are associated with LQT3 and cause increased late Na^+^ current when expressed in heterologous cells (14). CaM overexpression readily diminished late Na^+^ currents for these mutants, further supporting a functional role of apoCaM in tuning late Na current (14). Beyond the IQ motif, some reports also proposed a Ca^2+^-CaM interaction with the III–IV linker (15–17) — that the III–IV linker and C-terminal domain interaction is enhanced by CaM (13) — and a role for a Ca^2+^-dependent interaction in Na_V_1.5 inactivation (15–17). We found, however, no effect of Ca^2+^-CaM on Na_V_1.5 channel inactivation (13, 18). These findings all hint at CaM as a major regulator of Na_V_1.5 channel function, with disruption of CaM interaction with the C-terminal domain potentially unveiling pathogenic late Na current, predicted to trigger cardiac instability. Even so, the functional manifestation of this regulation and its potential pathophysiological role in the context of cardiomyocytes has never been established. This gap in understanding is critical, as ion channel function and regulation are exquisitely tuned by a rich repertoire of modulatory proteins that may be present in cardiomyocytes but not in heterologous expression systems.

Knock-in mice are valuable in the assessment of physiology and pathophysiology in cardiomyocytes, although — compared with humans — there are acknowledged differences in the ion channel profile and electrophysiologic properties, especially of repolarizing currents. Here, we developed an alternative approach of using transgenic mice expressing doxycycline-inducible, tissue-specific, FLAG epitope–tagged tetrodotoxin-sensitive (TTX-sensitive) Na_V_1.5 channels. Since the expression of these channels is inducible and cardiac specific, there is a low likelihood of developmental or other compensatory mechanisms affecting the phenotype. Moreover, from the same cardiomyocyte or animal, we can assess the electrophysiological characteristics of the endogenous and transgenically expressed channels. These channels, when expressed in cardiomyocytes, demonstrated electrophysiologic properties similar to endogenous channels. In this background, we created additional transgenic mice expressing alanine substitutions for IQ motif (IQ/AA), which prevents CaM binding to the C-terminus of Na_V_1.5. We found that the Na_V_1.5 IQ/AA mutant channels in cardiomyocytes did not appreciably increase late Na^+^ current, hinting at the presence of endogenous protective mechanisms. By comparison with cardiomyocytes, the Na_V_1.5 IQ/AA channels exhibit strong late Na^+^ current when expressed heterologously. To determine regulatory factors that prevent late Na^+^ current for IQ/AA mutant ch, we considered fibroblast growth factor homologous factors (FHFs), which are within the Na_V_1.5 proteomic subdomain in the heart, can bind to the C-terminal domain of Na_V_1.5, and have been implicated in the regulation of late Na^+^ current (11, 12, 19–21). We found that FGF13, the predominant cardiac isoform in rodents (22), diminished late current of the IQ/AA mutant, suggesting that endogenous FHFs may serve to prevent late Na^+^ current in cardiomyocytes. These findings highlight a protective role for endogenous regulatory proteins in preventing pathogenic late Na^+^ current in the heart, a potential pathway that may be leveraged to devise next-generation antiarrhythmics.

## Results

### Generation of inducible, cardiac-specific Na_V_1.5 transgenic mice.

We created transgenic mice with *tetO*–3XFLAG epitope–tagged, TTX-sensitive (C374Y) transgenic mice (designated pseudo-WT, pWT) and *tetO*–3XFLAG epitope–tagged, TTX-sensitive IQ/AA transgenic mice (designated IQ/AA) (Figure 1B). The channel was engineered to be TTX sensitive with the substitution of C374Y (23), as Na_V_1.5 channels are relatively resistant to TTX compared with neuronal and skeletal muscle isoforms. The *tetO*-Na_V_1.5 mice were crossed to transgenic mice expressing a reverse transactivator (rtTA) controlled by the cardiac-specific α-MHC promoter (Figure 1B). The bitransgenic tetracycline-regulated system enabled robust expression of the FLAG-tagged transgenic Na_V_1.5 channels only when both transgenes and doxycycline were present.

Expression of the FLAG-tagged transgenic Na_V_1.5 channels in ventricles was assessed using an anti-FLAG antibody. In cardiomyocytes isolated from nontransgenic (NTG) mice, no signal was detected. Both the FLAG-tagged pWT and FLAG-tagged IQ/AA Na_V_1.5 channels were detected in ventricular myocytes isolated from bitransgenic mice (Figure 1C; see complete unedited blots in the supplemental material; https://doi.org/10.1172/jci.insight.141736DS1). For most experiments, mice were fed doxycycline for 1–5 days. In 1 of the 2 IQ/AA lines, doxycycline was not required for expression, although expression of rtTA was required, likely due to a low basal binding of rtTA protein to the *Tet* operator sequences (so-called leak) (24). Confirming the expression of the transgenic Na_V_1.5, immunofluorescence staining of fixed cardiomyocytes from pWT and IQ/AA mutant transgenic mice with an anti-FLAG antibody showed a membrane distribution consistent with that of endogenously expressed Na_V_1.5 channels (Figure 1D).

The total peak current in the transgenic cardiomyocytes is the sum of the endogenous Na^+^ currents and the transgenic Na_V_1.5 currents. Endogenous cardiac Na_V_1.5 are largely resistant to TTX. Consistent with this observation, 20 nM TTX had no effect on peak endogenous Na^+^ currents in cardiomyocytes isolated from nontransgenic animals (Figure 1, E and H). In cardiomyocytes isolated from pWT or IQ/AA transgenic mice, in contrast, 20 nM TTX reduced the peak Na^+^ current, implying that the transgenic channels were expressed, inserted in the membrane, and were functional (Figure 1, F–H, and Supplemental Figure 1, A, B, D, and E). The difference between the total current and the remaining current after TTX infusion represents the current carried by the transgenic pWT or IQ/AA channels (Supplemental Figure 1, C–F). The mean fraction of TTX-sensitive current, which can be attributed to the transgenic Na^+^ channels, was 69% and 71% for pWT and IQ/AA, respectively (Figure 1I).

### Late Na^+^ current is not increased in IQ/AA cardiomyocytes.

**Figure 2:**
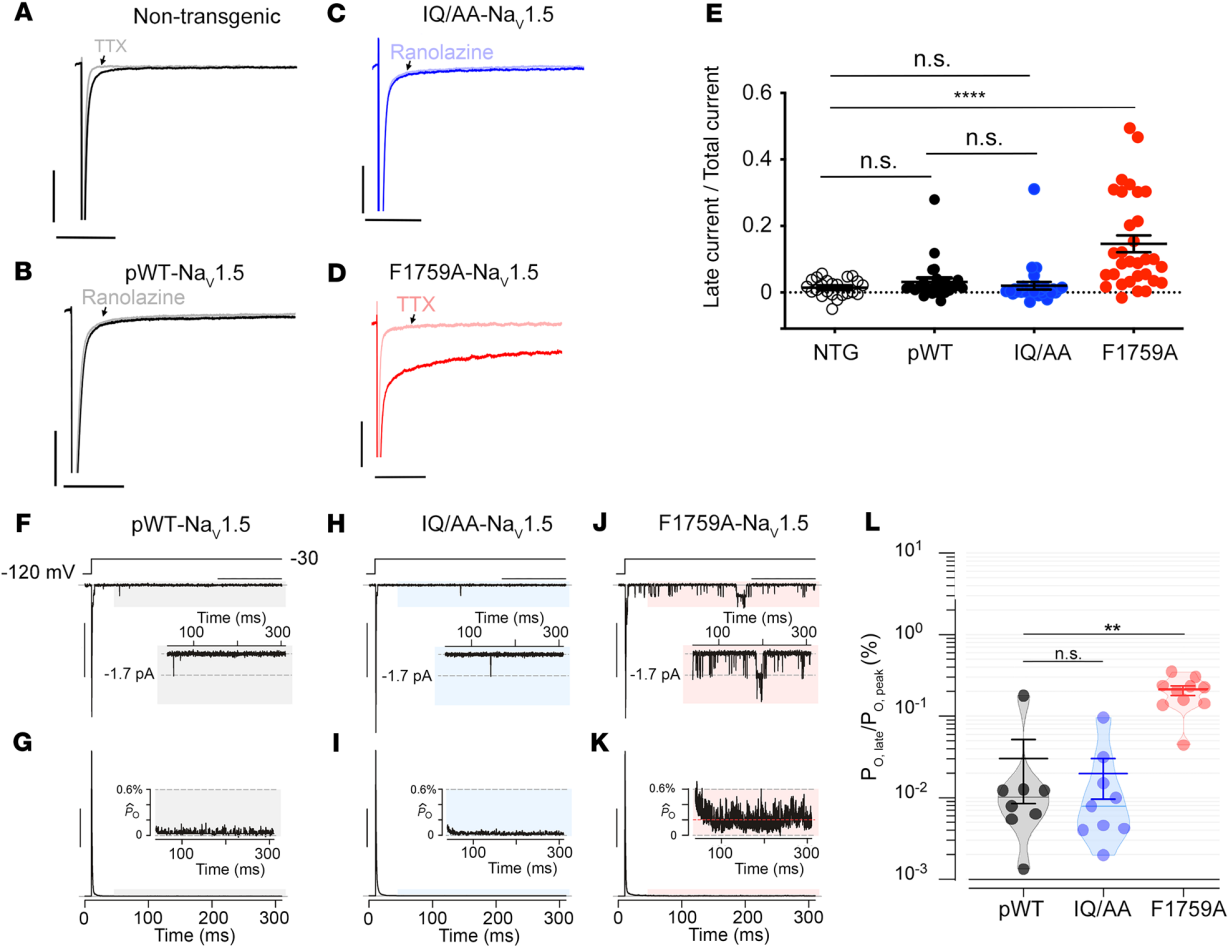
Late Na^+^ current is not increased in cardiomyocytes expressing IQ/AA Na_V_1.5. (**A–D**) Exemplar whole cell Na^+^ current traces of ventricular cardiomyocytes isolated from nontransgenic, pWT, IQ/AA, and F1759A mice. Experiments designed to assess late Na^+^ current using a 190 ms depolarization from a holding potential of –110 to –30 mV in the absence and presence of 500 μM ranolazine or 40 μM TTX; intracellular solution contained 5 mM Na^+^ and extracellular solution contained 100 mM Na^+^. Horizontal scale bars: 50 ms; vertical scale bars: 10 pA/pF. (**E**) Graph of fraction of late Na^+^ current normalized to peak Na^+^ current. Mean ± SEM, ****P < 0.0001 by Kruskal-Wallis test with Dunn’s multiple comparison test. *n* = 23, 25, 29, and 31 cardiomyocytes from left to right. (**F**) Multichannel record from pseudo-WT myocyte shows rapid Na^+^ channel activation and inactivation, followed by a rare opening in the late phase, following 50 ms of depolarization (gray shaded region). Inset shows lone Na_V_1.5 opening to unitary current level (dashed line) in the late phase. Vertical scale bar: 10 pA; horizontal scale bar: 100 ms. (**G**) Normalized ensemble-average open probability relation computed from 50–80 stochastic records. Inset shows low levels of late *P*_O_ following 50 ms of depolarization. Vertical scale bar: 25% for normalized *P*_O_ (P_O_[t]/P_O,peak_). P_O_(t), time-dependent open probability; P_O,peak_ denotes the peak open probability. (**H** and **I**) Multichannel recordings of Na^+^ channels from IQ/AA mice show minimal late current similar to pWT myocytes. Format as in **F** and **G**. (**J** and **K**) Appreciable late Na^+^–channel openings were detected for F1759A mutant. Format as in **F** and **G**. (**L**) Graph of *P*_O_ normalized to peak *P*_O_. Mean ± SEM, **P < 0.001 by Kruskal-Wallis test, **P < 0.01 by Dunn’s multiple comparison test.

**Figure 3:**
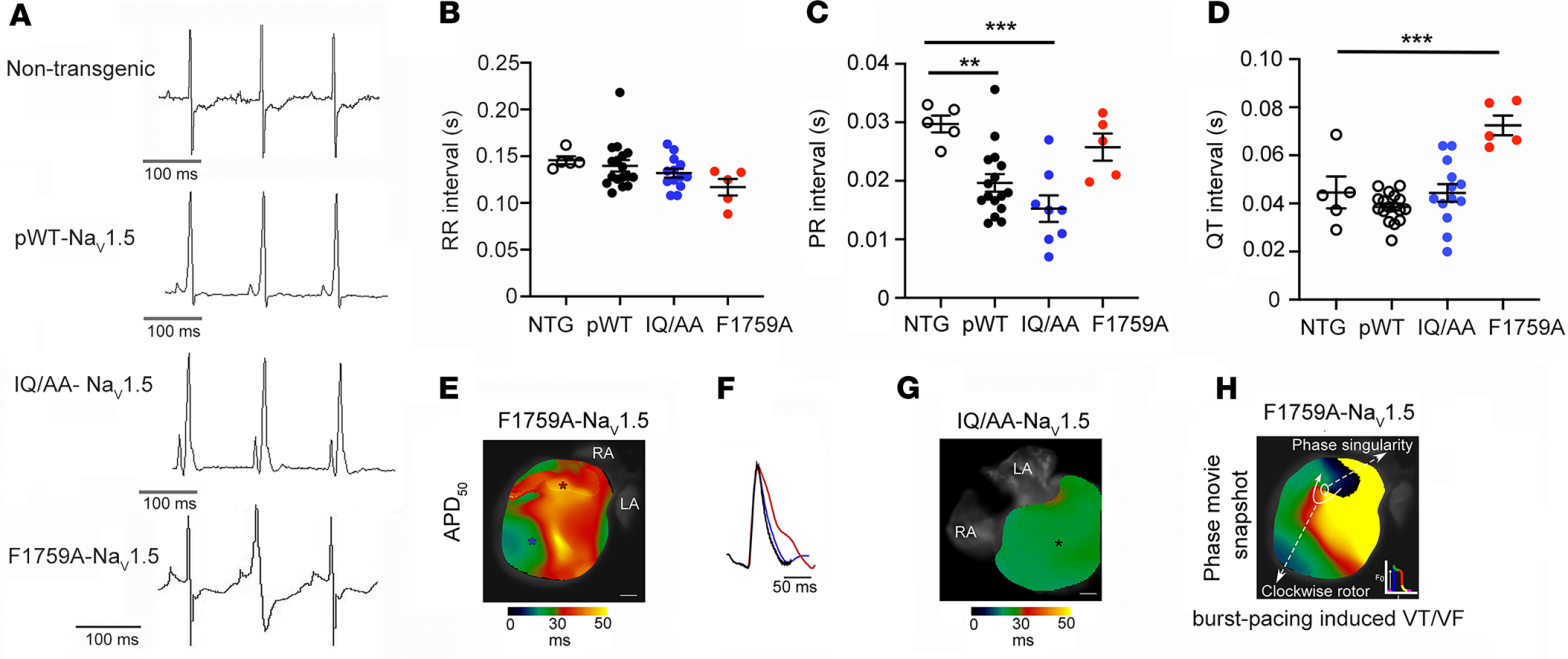
QT interval and ventricular repolarization is not prolonged in IQ/AA transgenic mice. (**A**) Representative limb-lead surface electrocardiograms of isoflurane-anesthetized littermate nontransgenic, pWT, IQ/AA, and F1759A transgenic mice. (**B–D**) Bar graphs of RR, PR, and QT intervals from isoflurane-anesthetized mice. Mean ± SEM. For RR interval, *P*= 0.12; for PR interval, *P* = 0.0004; for QT interval, *P* < 0.001 by 1-way ANOVA. **P < 0.01, ***P < 0.001 by Dunnett’s multiple comparison test. NTG, *n* = 5; pWT, *n* = 17; IQ/AA, *n* = 13; F1759A, *n* = 5. (**E–G**) Representative optical APD maps (**E** and **G**) and optical action potential tracings (**F**) from F1759A and IQ/AA mice. APD maps for F1759A-dTG were obtained after hyperkalemia-induced conversion to sinus rhythm. The circles in panels **E** and **G** mark the regions for which optical action potential tracings are displayed in **F**. Scale bar: 1 mm. Representative of 3 similar recordings. (**H**) Snapshot from phase movie of Langendorff-perfused F1759A-dTG hearts demonstrating rotor in the ventricle after burst pacing–induced ventricular arrhythmia. Representative of 3 similar experiments.

As late Na^+^ current is an inherent channel property that reflects alterations in channel inactivation, its magnitude is typically normalized to the peak current (5). This maneuver is essential, as it accounts for changes in peak current that may occur due to variability in channel expression in a transgenic model. The low magnitude of late current in relation to the peak (<1%), however, poses 2 experimental challenges for robust quantification. First, nonselective membrane leak or digitization artifacts could corrupt accurate resolution of small-amplitude late Na^+^ current. Second, when late current is amplified by large ionic gradients, the amplitude of the peak current becomes large and vulnerable to voltage-clamp artifacts. To overcome these limitations, we devised a protocol whereby the late current is measured using 100 mM Na^+^ and the peak current is measured using 3 mM or 5 mM Na^+^ in the extracellular solution. For the measurement of late current, we quantified the difference in currents during the final 10 ms of a 190-ms depolarization with 100 mM Na^+^ in the extracellular solution, before and after application of either TTX or ranolazine, a selective blocker of late Na^+^ current (Figure 2, A–D). After wash-out of ranolazine or TTX, peak current was measured with 3 mM Na^+^ in the extracellular solution in the absence and presence of 20 nM TTX for pWT or IQ/AA transgenic mice (Figure 1, E–G) or 3 mM lidocaine for F1759A transgenic mice (Supplemental Figure 2). Normalization of late Na^+^ current to the peak current yielded a late-current ratio enabling comparison between transgenic models. All recordings were performed with cesium fluoride (CsF) for patch stability and with BAPTA in the patch pipette to chelate intracellular Ca^2+^, thus querying the role of apoCaM.

The late-current ratio was not different between nontransgenic and pWT cardiomyocytes, implying that overexpression of TTX-sensitive Na_V_1.5 in mice does not alter the ratio of late-to-peak current (Figure 2E). Similarly, the late-current ratio was not increased in the IQ/AA mice compared with either the late-current ratio in NTG or pWT mice (Figure 2E). This finding was unexpected, since LQT3 mutations that disrupt CaM interaction cause increases in late current when measured in a heterologous expression system (14), and it contrasts with our findings from F1759A lidocaine-resistant transgenic channels in which the late-current ratio was markedly increased compared with nontransgenic, pWT, and IQ/AA mice (Figure 2, D and E).

To further scrutinize late Na^+^ current in the pWT, IQ/AA, and F1759A transgenic models, we undertook cell-attached multichannel recordings to directly measure late-channel openings (25, 26). A 300 ms depolarizing pulse is used to elicit channel openings, as evident in a patch from pWT transgenic mice (Figure 2F). Rapid activation followed by inactivation of multiple channels within the patch result in stacked channel openings reflecting near-macroscopic peak current. As late openings still occur to the unitary current level, they can be easily distinguished from instrument noise and baseline level. For channels from pWT myocytes, we observed sparse late-channel openings, consistent with near-complete inactivation (Figure 2F, shaded area and inset). For each patch, we obtained 50–100 stochastic traces to compute an ensemble average current, which was subsequently divided by the unitary current level (*i*) to obtain *NP*_O_, where N represents the number of channels in the patch and P_O_ represents the open probability.. To determine late current, we normalize *NP*_O_ trace by its peak value to obtain a normalized *P*_O_ waveform for each patch (Figure 2G) and compute the mean value following 50 ms of depolarization (Figure 2L). Similar to pWT, multichannel recordings from IQ/AA transgenic cardiomyoyctes also showed minimal late-channel openings, as evident from exemplar trace (Figure 2H) and ensemble average (Figure 2I). By comparison, Na^+^ channels from F1759A cardiomyocytes showed incomplete inactivation with frequent channel openings even 50 ms following depolarization (Figure 2, J and K). The ratio of late-to-peak open probability was significantly increased for F1759A channels but not for IQ/AA channels compared with the control channels (Figure 2L). These findings contrast with reports from studies of IQ/AA mutant channels expressed in HEK cells where the IQ/AA mutation results in a nearly 7-fold increase in late current (13).

### Repolarization not prolonged in IQ/AA transgenic mice.

To assess whether disruption of apoCaM binding to Na_V_1.5 affected electrophysiological properties of the heart in vivo, we performed electrocardiographic (ECG) analyses on the mice. Increased late Na^+^ current from Na_V_1.5 manifests as a prolonged QT interval on the ECG and is the basis for LQT3 in humans (3, 4). Compared with NTG mice, the heart rates (R-R interval) in pWT and IQ/AA mice were unchanged (Figure 3, A and B). The PR interval, which represents the conduction time from the sinus node through initial activation of the ventricles via the atrioventricular (AV) node was shorter in both pWT and IQ/AA transgenic mice (Figure 3, A and C), consistent with previously created transgenic mouse models with WT Na_V_1.5 overexpression (27). The PR was not shortened in the F1759A-Na_V_1.5 mice (Figure 3C) compared with NTG controls, perhaps reflecting the balance between Na^+^ channel overexpression and prolonged repolarization. In some patients with LQT3, prolonged AV conduction time and AV block has been reported (28). The QT interval in IQ/AA mice was not increased compared with NTG or pWT (Figure 3, A and D), which was unexpected in light of reported LQT3 mutations (and corresponding increased QT interval) that disrupt CaM interaction. In comparison, expression of mutant F1759A-Na_V_1.5 channels that enhance the late Na^+^ current in a heterologous system caused prolonged QT interval (Figure 3, A and D) (5).

Epicardial surface optical voltage mapping of the anterior surface of Langendorff-perfused IQ/AA and F1759A-NaV1.5 hearts was used to assess ventricular repolarization (Figure 3, E–G). As we have done previously (29), the Langendorff apparatus–mounted hearts were perfused with a hyperkalemic solution to terminate the spontaneous arrhythmias in order to elucidate the underlying electrophysiologic substrate. Thereafter, a normokalemic solution was infused, and the ventricular APD was assessed. In the F1759A mutant mice, we observed APD prolongation (Figure 3, E and F). As we have previously described for the atria (5, 30), there was considerable heterogeneity in the APD, likely caused by the variable expression of the F1759A-Na^+^ channels. In contrast, the IQ/AA transgenic mice had uniformly normal ventricular repolarization (Figure 3, F and G). We hypothesized that inhomogeneity of the prolonged ventricular APD can form the substrate for initiating and sustaining ventricular arrhythmias in F1759A mice. We optically acquired voltage maps of Langendorff-perfused hearts before and after burst pacing to induce ventricular arrhythmias, and we used phase mapping to quantify rotational reentry dynamics, including phase singularity points. The F1759A transgenic hearts were readily inducible into rotational reentry–dependent ventricular tachycardia/fibrillation (Figure 3H), which was not observed in the IQ/AA hearts. Consistent with this finding, spontaneous premature ventricular contractions were frequently observed on 12-lead ECG of F1759A mice but not in IQ/AA mice. Taken together, the absence of QT interval and cardiac action potential prolongation is consistent with the lack of increased late current in the IQ/AA mice, suggesting that in vivo loss of CaM binding can be fully compensated in mice.

### FHF and CaM in complex with Na_V_1.5 in cardiomyocytes.

**Figure 4:**
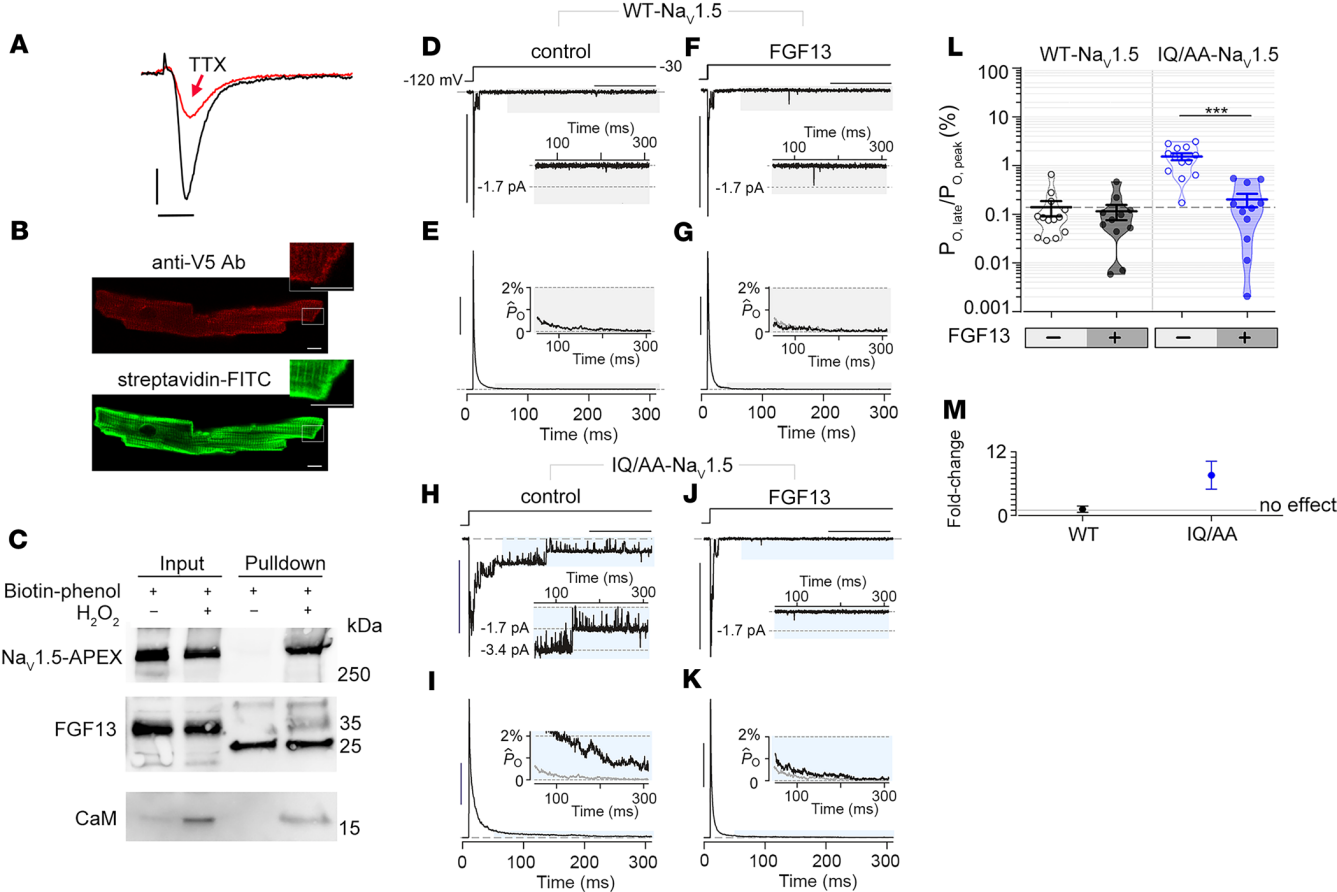
Expression of FGF13 reduces late Na^+^ current in IQ/AA Na_V_1.5. (**A**) Exemplar whole cell Na^+^ currents recorded from cardiomyocytes of TTX-sensitive Na_V_1.5-V5-APEX2 transgenic mice, before (black trace) and after (red trace) 20 nM TTX. Representative of 3 similar recordings. Vertical scale bar: 5 pA/pF; horizontal scale bar: 5 ms. (**B**) Immunofluorescence of cardiomyocytes isolated from mice expressing Na_V_1.5-V5-APEX2 exposed to biotin-phenol and H_2_O_2_. Staining is with anti-V5– and –Alexa 594–conjugated secondary antibodies (upper) and streptavidin-conjugated Alexa 488. Scale bar: 5 μm. (**C**) Immunoblots of biotin-labeled proteins from cardiomyocytes of Na_V_1.5-APEX2 mice. Na_V_1.5, FGF13, and CaM are detected in streptavidin pull-down. Blots are representative of 2 independent experiments. (**D** and **E**) Multichannel recordings show minimal late Na^+^–channel openings for WT-Na_V_1.5 expressed in HEK293 cells. Format as in Figure 2, F and G. Horizontal scale bar: 100 ms; vertical scale bar: 10 pA (**D**) and 25% for normalized *P*_O_ (*P*_O_[t]/*P*_O,peak_) (**E**). (**F** and **G**) Late current of WT-Na_V_1.5 is unaffected by FGF13 overexpression. Same format as in **D** and **E**. (**H** and **I**) IQ/AA Na_V_1.5 mutant channel showing enhanced late-channel openings compared with WT-Na_V_1.5 in HEK293 cells. Same format as in **D** and **E**. (**J** and **K**) FGF13 coexpression with IQ/AA Na_V_1.5 reverses the increase in late current to WT levels. Same format as in **D** and **E**. (**L**) Dot plot summary of *P*_O,late_ for WT-Na_V_1.5 and IQ/AA Na_V_1.5 in the presence and absence of FGF13. Mean ± SEM, ***P < 0.0001 by Kruskal-Wallis test with Dunn’s multiple comparison test. (**M**) Graph shows fold-change in *P*_O,late_ for WT-Na_V_1.5 and IQ/AA Na_V_1.5 by FGF13. Mean ± SEM, computed from aggregate data in **L**.

We adapted for application to cardiomyocytes the ascorbate peroxidase 2 (APEX2) proximity labeling method, originally developed for the identification of the mitochondrial proteome (31, 32). We recently used this approach to obtain a comprehensive proteome of Ca_V_1.2 in cardiomyocytes (33). We generated transgenic mice with doxycycline-inducible, cardiomyocyte-specific expression of TTX-sensitive Na_V_1.5 proteins with APEX2 and a V5 epitope conjugated to the N-terminus, enabling biotin labeling of proteins within an approximately 20 nm radius. Fusing APEX2 to Na_V_1.5 did not affect Na_V_1.5 subcellular localization and function, as assessed by cellular electrophysiology and anti-V5 antibody immunofluorescence (Figure 4, A and B). Incubating isolated ventricular cardiomyocytes with a solution containing biotin-phenol induced robust biotinylation of proteins at the sarcolemma, at the intercalated disk, and in a striated z-disk pattern, coinciding with the pattern of transgenic Na_V_1.5 expression (Figure 4B). Western blots show the biotinylation and streptavidin purification of both CaM and FGF13 (Figure 4C; see complete unedited blots in the supplemental material), confirming prior studies showing FGF13 and Na_V_1.5 interact (22, 34) and consistent with known cellular distribution of FHF13 in transverse tubules and the sarcolemma (35). As FHF proteins have been shown to modify multiple aspects of Na_V_ channel inactivation and to reduce late current in heterologously expressed C-terminal–deleted Na_V_1.5 at residue 1885 — which deletes the C-terminus, including the IQ-motif (21) — we reasoned that FGF13 may be in part responsible for attenuating late current of IQ/AA mutant in vivo. FGF13 binds to the C-terminus of Na_V_1.5 with an isothermal calorimetry–determined (ITC-determined) affinity of 16.6 nM (Supplemental Figure 3), which is approximately 10-fold higher than the affinity of FGF12B (11) — the FHF isoform expressed in human cardiomyocytes.

### Expression of FGF13 reduces late current caused by IQ/AA but not F1759A mutations.

Since HEK cells lack endogenous FHFs, we tested whether FGF13 overexpression in these cells might recapitulate the differential penetrance of late Na^+^ current that we observed for the IQ/AA versus F1759A mutant in cardiomyocytes. As a control, we quantified late-channel openings from WT Na_V_1.5 channels in the presence and absence of FGF13 using multichannel cell-attached recordings. At baseline, WT channels exhibited minimal late-channel openings (Figure 4, D and E), and FGF13 coexpression had no appreciable effect (Figure 4, F, G, L, and M). Expression of Na_V_1.5 IQ/AA mutant alone revealed late-channel openings (Figure 4, H and I) with an 11-fold increase in late current (Figure 4, L and M), consistent with previous studies (13). To mimic the colocalization of FHF with Na_V_1.5 in cardiomyocytes, we coexpressed FGF13 with Na_V_1.5 IQ/AA mutant. This maneuver resulted in a complete reversal of late current to WT levels (Figure 4, J–M), suggesting that the addition of FGF13 eliminated late current when the IQ motif was completely ablated and reminiscent of the absence of late Na^+^ current in IQ/AA transgenic cardiomyocytes in which FGF13 is present (Figure 2). These findings suggest that endogenous FGF13 in cardiomyocytes may serve a protective function by preventing pathogenic late Na^+^–channel openings.

**Figure 5:**
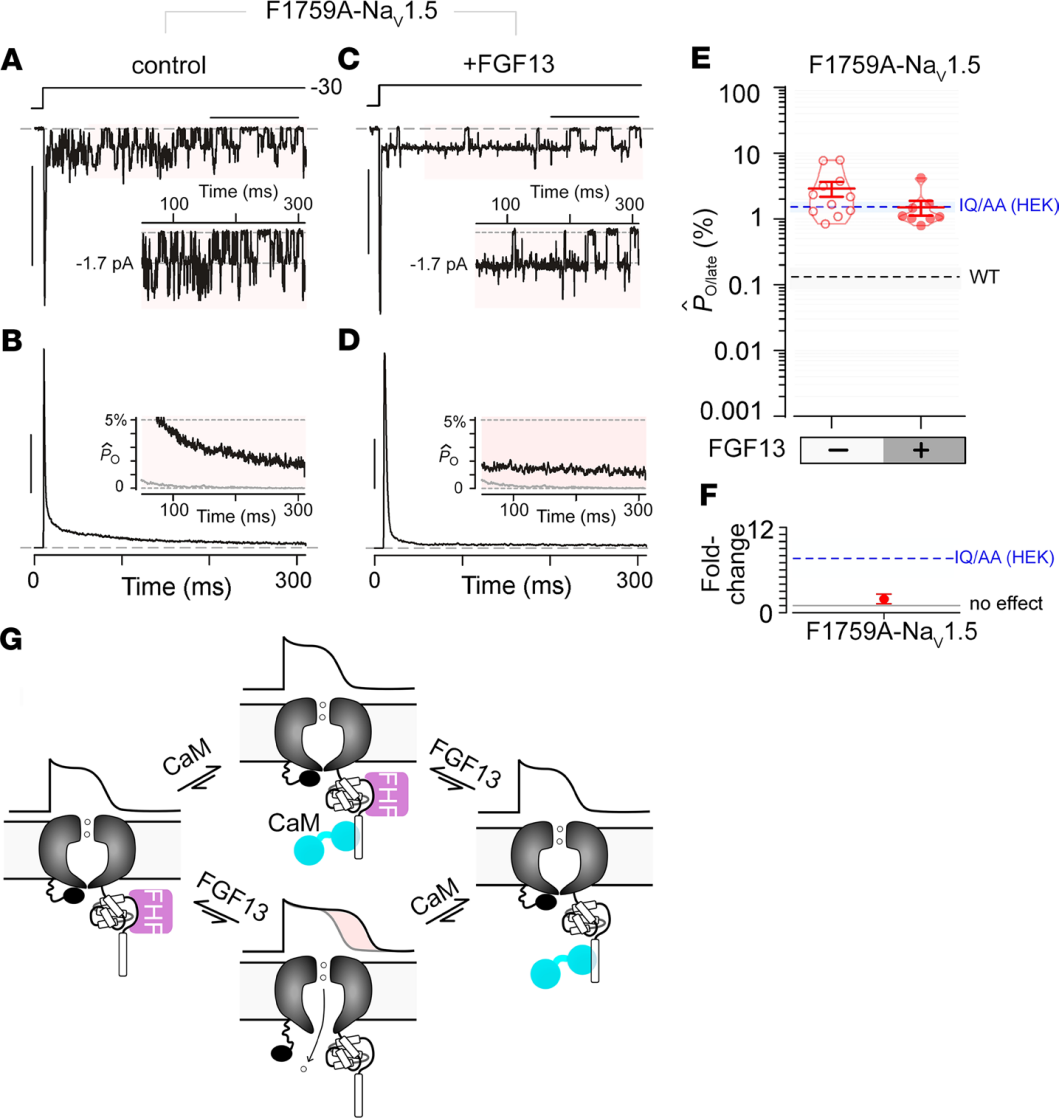
Late Na^+^ current for F1759A-Na_V_1.5 is minimally perturbed by FGF13. (**A** and **B**) Multichannel recordings show high late Na^+^ current for F1759A-Na_V_1.5 mutant when expressed in HEK293 cells. Format as in Figure 2, F and G. Horizontal scale bar: 100 ms; vertical scale bar: 10 pA (**A**) and 25% for normalized *P*_O_ (*P*_O_[t]/*P*_O,peak_) (**B**). (**C** and **D**) FGF13 coexpression with F1759A-Na_V_1.5 mutant. Horizontal scale bar: 100 ms; vertical scale bar:10 pA (**C**) and 25% for normalized *P*_O_ (*P*_O_[t]/*P*_O,peak_) (**D**). (**E** and **F**) Population data confirm minimal change in late *P*_O_ for F1759A-Na_V_1.5 with FGF13. Format as in Figure 4, L and M. Open probability of late Na^+^ current from heterologously expressed IQ/AA Na_V_1.5 (without FHF13) is shown by the dashed blue line. (**G**) Schematic depicting late Na^+^ current in the absence of both FGF13 and CaM binding to the C-terminal domain of Na_V_1.5. Late Na^+^ current is not present when either FHF or CaM binds to the C-terminal domain of Na_V_1.5.

In cardiomyocytes, F1759A mutants exhibit a substantial late Na^+^ current that causes cardiac arrhythmias in mice, raising the possibility that these channels are less sensitive to endogenous protective mechanisms. To test this possibility, we expressed the Na_V_1.5 F1759A channels in HEK293 cells and undertook multichannel recordings. Similar to cardiomyocytes, recombinantly expressed F1759A mutant display markedly increased late openings (Figure 5, A and B), as previously reported (36). Interestingly, the amount of late current for F1759A is similar to the IQ/AA mutant in the absence of FHFs. In contrast to IQ/AA mutant, however, FGF13 overexpression exerted negligible effect on the late current of F1759A mutation (Figure 5, C–F), thus confirming the reduced sensitivity of these channels for FGF13. In all, the differential modulation of IQ/AA versus F1759A mutant by FGF13 further corroborates the emerging role of FGF13 in tuning a pathogenic late current of cardiac Na_V_ channels.

## Discussion

In cardiomyocytes, late Na^+^ current caused by delayed or incomplete inactivation of Na_V_1.5 channels underlies diverse cardiac disorders. Our previous studies using a transgenic Na_V_1.5 F1759A mutant channel illustrated the contribution of late current for cardiac disease pathogenesis (5, 30). Although diminutive compared with the peak Na^+^ current that initiates the cardiac action potential, the depolarizing late current counteracts repolarization, resulting in heterogenous action potential prolongation that may trigger atrial (5) or ventricular arrhythmias (Figure 3). Sustained Na^+^ influx also causes structural abnormalities such as those linked to dilated cardiomyopathy (3). In heterologous expression systems, CaM has emerged as an important regulator of late Na_V_1.5 current (13, 14). LQT3-linked and structure-guided mutations in the Na_V_1.5 carboxy-terminus that disrupt CaM interaction cause a marked increase in late Na^+^ current (13, 14) to levels comparable with those seen in the F1759A mutant. To probe for the functional relevance of CaM for late Na_V_1.5 current in cardiomyocytes, we generated a transgenic mouse model expressing IQ/AA mutant Na_V_1.5 channels with disrupted CaM binding. Surprisingly, we found that IQ/AA mutant did not increase late Na^+^ current in cardiomyocytes. These findings reveal the existence of endogenous protective mechanisms that counteract the increase in late current that occurs with loss of CaM binding (Figure 5G). Further biochemical and mechanistic analysis demonstrated that FGF13, a component of the Na_V_1.5 neighborhood in cardiomyocytes, fully reverses the late current of IQ/AA but minimally perturbs F1759A mutant in HEK293 cells.

CaM regulation of Na_V_ channels is multifaceted and isoform specific, although a vast majority of functional effects thus far have been deduced through heterologous expression of recombinant Na_V_ channels (13–15, 21, 37, 38). Both Ca^2+^-free and Ca^2+^-bound CaM interact with Na_V_1.5 channels with a high affinity (9–12, 15). The Ca^2+^/CaM-mediated effects of Na_V_1.5 have been variable, with some studies finding a depolarizing shift in steady-state inactivation properties depending on CaM interaction with the III–IV linker (15, 38). Prior analysis of endogenous Na_V_1.5 in guinea pig ventricular myocytes, however, revealed no Ca^2+^-dependent effects, leading us to focus here on effects of apoCaM (18). Indeed, loss of apoCaM preassociation to the Na_V_1.5 IQ motif results in a marked elevation of late current in heterologous systems caused by a structural uncoupling of the III–IV linker from the C-terminal domain (13, 21). Our present study, however, demonstrates that this is not the case for CaM-deficient IQ/AA mutant channels in mouse cardiomyocytes. Thus, the manifestation of CaM regulation of Na_V_ channels diverges considerably in the native setting compared with heterologous systems.

The absence of both apoCaM- and Ca^2+^-CaM–dependent modulation of Na_V_1.5 in cardiomyocytes raises new questions regarding the functional role of CaM for these channels. This is critical, as human mutations in the CaM binding interface of Na_V_1.5 are linked to LQT3 (14), although augmentation of late Na^+^ current is likely not the main pathophysiological mechanism responsible for the LQTS phenotype observed in patients with CaM mutations (39). One possibility is that CaM plays a redundant role also served by FHF to ensure fail-safe inactivation (21), a critical factor for proper cardiac function (Figure 5G). However, if FHF and CaM cooperatively ensure fail-safe inactivation, why should the human mutation Q1909R (40), which decreases CaM affinity, lead to LQT3 and arrhythmias in humans? No apparent phenotype would be expected based upon the findings for the IQ/AA mutant in mouse cardiomyocytes. There are several possible explanations. (a) Mice and humans express different FGF isoforms; mice express FGF13, and humans express predominantly FGF12 (35). The affinity of FGF13, ~16 nM, for the Na_V_1.5 C-terminal domain (Supplemental Figure 3) is approximately 10-fold higher relative to FGF12 (11). Thus, mice may be more “protected” than humans to CaM depletion–induced Na_V_1.5-mediated arrhythmias because of expression of FGF13 rather than FGF12. Studies of inducible pluripotent stem cell–derived differentiated cardiomyocytes may further delineate the role of FGF12 in tuning CaM-deficient Na_V_1.5 channels in humans. (b) FHF expression and interactions with Na_V_1.5 may be tuned by transcriptional or posttranslation modifications (34, 41), which unveils latent CaM-dependent modulation. Perhaps the phenotype of Q1909R becomes manifest only in specific circumstances, which may not be apparent in healthy sedentary laboratory animals. For instance, a bevy of other cellular factors and conditions have been shown to upregulate late Na^+^ current(42, 43), including heart failure (44), hypoxia (45, 46), reactive oxygen species (45), CaMKII-dependent phosphorylation (47, 48), and altered interaction with other regulatory proteins (49, 50). It is possible that some of these processes may be linked to FHF modulation of Na_V_1.5. For example, CaMKII-dependent phosphorylation of the Na_V_ carboxyl-tail has been shown to disrupt FHF interaction (34). (c) Finally, FHF-CaM regulation of Na_V_ channels can be synergistic or antagonistic depending on the physiological setting. For skeletal muscle Na_V_1.4 channels, CaM regulation manifests as rapid Ca^2+^-dependent inhibition of the peak current (18). FHF coexpression with Na_V_1.4 antagonizes Ca^2+^/CaM-dependent feedback of these channels (51). This functional plasticity may help ensure that Na^+^ influx is precisely tuned to match varied physiological demands.

The relative insensitivity of F1759A mutant for FGF13 could be predicted by the presence of late current in cardiomyocytes. Structurally, F1759 residue is located within the transmembrane region in a central cavity on the intracellular side of the selectivity filter (7). This location is within the membrane, rather than on the C-terminal domain, which is the primary FHF binding site (11). As such, we expect intact FHF binding to the F1759A mutant. Na_V_ channels in Purkinje neurons exhibit a resurgent current that results from open-state block by intracellular endogenous proteins, including FGF14 and the cytosolic domain of the Na_V_ β4 subunit (52). Interestingly, such endogenous open-channel blocking proteins antagonize lidocaine action, suggesting that the 2 sites may be coupled (53).

Future studies that knock out FGF13 in the IQ/AA transgenic model will help ascertain the precise contribution of FGF13 in attenuating the late current of FGF13. In our multichannel recordings, the peak-normalized late open probability of WT Na_V_1.5 channels bound to FGF13 in HEK cells are nearly an order of magnitude higher than that of the native cardiac setting. Careful scrutiny of late-channel openings of F1759A mutant in cardiomyocytes reveal a “late-scattered” phenotype, while FHF coexpression with F1759A mutant in HEK cells show “burst mode” openings — 2 distinct mechanisms for late current (42). These differences in channel gating behavior suggest that there may be as-yet-unidentified regulatory proteins in addition to FHF that may also be involved in vivo. Proteomic studies using the Na_V_1.5-APEX mice could help identify other regulatory proteins that diminish late current.

In all, our results reveal a surprisingly complete protection from late Na^+^ current in murine cardiomyocytes expressing Na_V_1.5 devoid of apoCaM binding. Heterologous expression studies suggest that the protection is due to FGF13, which is colocalized with Na_V_1.5 in murine cardiomyocytes. Leveraging endogenous mechanisms may furnish an alternative avenue for developing novel pharmacology that selectively blunts late Na^+^ current, a highly sought-after drug target (42, 43).

## Methods

### General experimental approaches.

All experimental procedures and analyses were performed in a blinded fashion. No data points, samples, or mice were excluded from the study.

### Mouse models.

The pWT, IQ/AA, F1759A, and V5-APEX2 lines were generated by fusing human heart Na^+^ channel α-subunit cDNA (hH1) (54) to a vector containing the modified murine α-MHC, tetracycline-inducible promoter gift of Jeffrey Robbins and Jeffrey Molkentin (University of Cincinnati, Cincinnati, Ohio, USA) (55, 56). *SCN5A* was engineered to be either TTX sensitive by inserting a C374Y mutation or lidocaine-resistant by insertion a F1759A mutation. A 3× FLAG epitope was ligated in-frame to the N-terminus. These mice, in a B6CBA/F2 hybrid background, were bred with cardiac-specific rtTA mice in a FVB/N background, obtained via Mutant Mouse Resource & Research Center (MMRRC) (24), to generate doxycycline-inducible transgenic mice. The V5 epitope and APEX2 cDNA (57, 58), created by gene synthesis, were conjugated to the N-terminus of human heart Na^+^ channel α-subunit. Both male and female mice were used in all experiments. Sex had no effect on the outcomes of any experiment.

### ECG analysis.

S.c. 4-lead electrocardiograms of isoflurane-anesthetized mice were performed using Emka ECG and recorded using IOX software (Emka Technologies). PR, RR, and QT intervals were measured manually using Ponemah 3 software (Data Sciences International).

### Isolation of cardiac myocytes from adult mice.

Mouse ventricular myocytes were isolated by enzymatic digestion using a Langendorff perfusion apparatus as previously described (5, 33, 59–62). Cardiomyocytes were isolated from 8- to 1- week-old nontransgenic and transgenic mice.

### Cardiomyocyte patch clamp recordings.

Experiments were performed at room temperature. Membrane currents from rod-shaped cells with clear striations that were not spontaneously contracting were measured by the whole-cell patch-clamp method using a MultiClamp 700B amplifier and pCLAMP software (Axon Instruments, Molecular Devices). The pipette resistance was 0.4–1.0 MΩ in order to minimize voltage clamp error. The cell capacitance currents were compensated. Series resistance was compensated at 60%. Liquid junction potential (–10 mV) was corrected. The leak current was subtracted using a P/4 protocol. The intracellular pipette solution contained (in mM; all solutions in the multichannel section below are from MilliporeSigma): 3 or 5 NaCl, 20 CsCl, 115 CsF, 10 HEPES, and 10 BAPTA (pH 7.4) titrated with CsOH. For late Na^+^ current determinations, the bath solution contained (in mM): 100 NaCl, 45 TEA-Cl, 10 HEPES, 1 MgCl_2_, 0.25 CaCl_2_, and 5 glucose (pH 7.4) titrated with CsOH. To determine late current, the cell membrane potential was held at –110 mM and stepped to –30 mV for 190 ms in the absence and in the presence of TTX (40 μM; nontransgenic and F1759A mice) or ranolazine (50 μM; pWT and IQ/AA mice). The mean value of the current during the last 10 ms of the 190-ms pulse was measured. The difference of these values was used as a measure of late Na^+^ current and later normalized to cell capacitance. The mean value of the late current was also normalized to the peak Na^+^ current for each cell. For measurement of peak Na^+^ current, the extracellular solution contained (in mM): 3 or 5 NaCl, 142 TEA-Cl, 10 HEPES, 1 MgCl_2_, 0.25 CaCl_2_, and 5 glucose (pH 7.4) titrated with CsOH. Peak transient currents in the F1759A mice were measured with 5 mM Na^+^ in both intracellular and extracellular solutions. For the cardiomyocytes isolated from F1759A mice, the fraction of transgenic current was assessed by applying 3 mM lidocaine as we have previously described (5). Peak transient currents in the cardiomyocytes isolated from the pWT and IQ/AA mice were measured with 3 mM Na^+^ in both intracellular and extracellular solutions by stepping the voltage from a holding potential of –110 mV to –30 mV. For these cardiomyocytes, the fraction of transgenic current was assessed by applying 20 nM TTX, which inhibits all mutant channels (IC_50_ < 1 nM) but has no effect on endogenous, nontransgenic channels. The current-voltage relationship of transgenic Na^+^ currents for the pWT and IQ/AA cardiomyocytes were quantified as the difference between no TTX and 20 nM TTX across a range of voltages, from a holding potential of –110 mV to 0 mV (Supplemental Figure 1).

### Multichannel analysis of late Na^+^ current.

Multichannel records were obtained in the on-cell configuration with either HEK293 cells or in cardiomyocytes. The pipette contained (in mM): 140 NaCl, 10 HEPES, and 0.5 CaCl_2_ at 300 mOsm adjusted with tetraethylammonium methanesulfonate, and pH 7.4 adjusted with tetraethylammonium hydroxide. To zero membrane potential, the bath contained (in mM): 132 K^+^-glutamate, 5 KCl, 5 NaCl, 3 MgCl, 2 EGTA, 10 glucose, and 20 HEPES at 300 mOsm adjusted with glucose, and pH 7.4 adjusted with NaOH. Data were acquired at room temperature using the integrating mode of an Axopatch 200A amplifier (Axon Instruments, Molecular Devices). Patch pipettes (3–10 MΩ) were pulled from ultra-thick–walled borosilicate glass (BF200-116-10; Sutter Instruments) using horizontal puller (P-97, Sutter Instruments), fire polished with a microforge (Narishige), and coated with Sylgard (Dow Corning). Elementary currents were low-pass filtered at 2 kHz with a 4-pole Bessel filter and digitized at 200 kHz with an ITC-18 unit (Instrutech), controlled by custom MATLAB software (Mathworks). For each pulse, we obtained P/8 leak pulses. Leak subtraction was performed using an automated algorithm, which fit the kinetics of the leak current or the capacitive transient with convex optimization with L1 regularization of the following the objective function:

 (Equation 1)
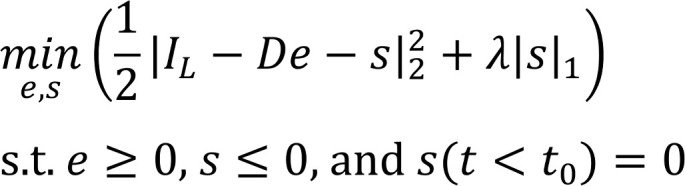


Here, *I*_L_ is the leak waveform that is to be fit as a sum of exponentials; *D* is a matrix composed of a library of exponential functions with various time constants; *e* is a sparse vector composed of the amplitude of each exponentials; *s* is a vector representing an estimate of negative outliers from the baseline that correspond to actual channel openings; and λ is a penalty term that controls the importance of regularization. For most fits, λ was set to be 0.25. Following leak subtraction, the unitary current for each patch was estimated using an amplitude histogram. Each stochastic trace was subsequently idealized. The ensemble average from 50 to 100 stochastic traces was computed for each patch and normalized to the peak current. The average late current for each patch (*R*_persist_) was computed as the average normalized *P*_O_ following 50 ms of depolarization.

### Cell culture and transfection.

Human Na_V_1.5 pGW corresponds to clone M77235.1 (GenBank). To construct IQ/AA mutation, we perform overlap-extension PCR and ligated the mutated PCR product into the Na_V_1.5 WT pGW vector using KpnI and XbaI restriction sites. The F1759 plasmid was constructed as previously described. Human FGF13/FGF13 plasmid corresponding to NM_004114.5 (GenBank) was cloned into the pcDNA3 vector following PCR amplification using EcoRI and XbaI. For whole-cell patch clamp experiments, HEK293 cells (ATCC) were cultured on 6-cm plates, and channels were transiently transfected by the Ca^2+^ phosphate method as previously described. For Na_V_1.5 WT and IQ/AA channels, we transfected 4–8 μg of the α-subunit with 4 μg of YFP and 1 μg of t-antigen to boost expression. For experiments evaluating the effect of FHF, we transfected FGF13-pcDNA3 at 1:1 ratio as the α-subunit.

### Proximity labeling biotinylation.

Proximity labeling was performed as described (33, 58). Isolated ventricular cardiomyocytes were incubated in labeling solution with 0.5 μM biotin-phenol (Iris Biotech) for 30 minutes. During the final 10 minutes of labeling, 1 μM isoproterenol (MilliporeSigma, I5627) was added. To initiate labeling, H_2_O_2_ (MilliporeSigma, H1009) was added to a final concentration of 1 mM for 1 minute. Exactly 1 minute after H_2_O_2_ treatment, the labeling solution was decanted and cells were washed 3 times with cold quenching solution containing (in mM) 10 sodium ascorbate (VWR, 95035-692), 5 trolox (MilliporeSigma, 238813), and 10 sodium azide (MilliporeSigma, S2002). After cells were harvested by centrifugation (800*g* for 1 minute at room temperature), the quenching solution was aspirated and the pellet was flash-frozen and stored at –80°C until streptavidin pull-down.

The cells were lysed with a hand-held tip homogenizer in a solution containing (in mM), 50 Tris (tris[hydroxymethyl]aminomethane), 150 NaCl, 10 EGTA, 10 EDTA, 1% Triton X-100 (v/v), 0.1% SDS (w/v), 10 sodium ascorbate, 5 trolox, and 10 sodium azide, phosphatase inhibitors (MilliporeSigma, 4906845001), protease inhibitors (MilliporeSigma, 4693159001), calpain inhibitor I (MilliporeSigma, A6185), and calpain inhibitor II (MilliporeSigma, A6060). Biotin labeling of the samples was confirmed by Western blotting with streptavidin-HRP (MilliporeSigma, RABHRP3). Proteins were prepared as previously described (63). Proteins were precipitated with trichloroacetic acid (TCA; MilliporeSigma, T9159) and then centrifuged at 21,130*g* at 4°C for 10 minutes. The pellet was washed with –20°C cold acetone (MilliporeSigma, 650501), vortexed, and centrifuged at 21,130*g* at 4°C for 10 minutes. Following centrifugation, acetone was aspirated and the pellet was acetone washed again 3 more times. After the last washing step, the pellet was resuspended in: 8M urea, 100 mM sodium phosphate (pH 8), 100 mM NH_4_HCO_3_, and 1% SDS (w/v) and was rotated at room temperature until fully dissolved. Resuspended proteins were centrifuged at 21,130*g* at room temperature for 10 minutes, and the cleared supernatant was transferred to a new microcentrifuge tube. To reduce disulfides, 10 mM TCEP-HCl (Thermo Fisher Scientific, PG82089) in Milli-Q water titrated to pH 7.5 with NaOH was added. To alkylate free Cys, freshly prepared 400 mM iodoacetamide (Thermo Fisher Scientific, 90034) stock solution in 50 mM ammonium bicarbonate was added to the supernatant to a final concentration of 20 mM, immediately vortexed, and incubated in the dark for 25 minutes at room temperature. After alkylation, freshly prepared DTT (dithiothreitol) stock solution was added to 50 mM final concentration to quench alkylation. Water was added to each sample to reach a final concentration of 4 M urea and 0.5% (w/v) of SDS.

For each sample, a 100 μL suspension of streptavidin magnetic beads (Thermo Fisher Scientific, 88817) was washed twice with 4M urea, 0.5% SDS (w/v), 100 mM sodium phosphate pH 8. The liquid was aspirated and the beads were added to each ~1 mg sample, thereafter diluting each sample with an equal amount of water to reach a final concentration of 2M urea, 0.25% SDS (w/v), and 50 mM sodium phosphate (pH 8) during pull-down. The tubes were rotated overnight at 4°C. Following streptavidin pull-down, the magnetic beads were washed 3 times with 4M urea, 0.5% SDS (w/v), and 100 mM sodium phosphate (pH 8) and washed 3 times with the same buffer without SDS. The beads were transferred to new tubes for the last wash step.

### Immunoblots.

Cardiomyocytes were homogenized in a 1% Triton X-100 buffer containing (in mM): 50 Tris-HCl (pH 7.4) 150 NaCl, 10 EDTA, 10 EGTA, and protease inhibitors. The lysates were incubated on ice for 30 minutes, centrifuged at 21,900*g* at 4°C for 10 minutes, and supernatants were collected. Proteins were size fractionated on SDS-PAGE, transferred to nitrocellulose membranes, and probed with anti-FLAG antibody (MilliporeSigma, A8592), a custom-made anti-FGF13 antibody (22), and an anti-CaM antibody (Millipore Sigma, 05-173). Image collection was performed with a CCD camera (Carestream Imaging), and quantification was performed using ImageQuant software.

### Immunofluorescence.

For proximity labeling, isolated cardiomyocytes were first exposed to biotin-phenol and H_2_O_2_ as described above. After quenching, the cells were fixed for 15 minutes in 4% paraformaldehyde, washed with glycine/PBS twice, treated with PBST (0.1% Triton X-100 [v/v] in PBS) for 5 minutes, and blocked with 3% BSA (w/v) in PBS for 1 hour. Indirect immunofluorescence was performed using a 1:500 anti-V5 antibody (Thermo Fisher Scientific, R960-25) and 1:200 Alexa 594–labeled goat-anti-mouse antibody (Thermo Fisher Scientific, A-11032), and 1:800 streptavidin Alexa Fluor 488 conjugate (Thermo Fisher Scientific, S32354). For immunofluorescence without proximity labeling, isolated cardiomyocytes were fixed for 15 minutes in 4% paraformaldehyde. Indirect immunofluorescence was performed using either a 1:200 rabbit anti-FLAG antibody (MilliporeSigma, F7425) or anti-NaV1.5 antibody (Alomone, ASC-005), and 1:400 FITC-labeled goat anti-rabbit antibody (Thermo Fisher Scientific, A-11034). Images were acquired using a confocal microscope.

### Optical mapping protocol and data processing.

Mice were heparinized, and isolated hearts were perfused via a Langendorff apparatus with warm oxygenated Krebs-Henseleit buffer (pH 7.4; 95% O_2_, 5% CO_2_, 36°C–38°C). The hearts were also superfused in a glass chamber filled with Tyrode. One AgCl wire was attached to the metal aortic cannula, and another AgCl wire was positioned near the surface of the heart to record an ECG. Blebbistatin (5–10 μM; Tocris) was perfused to reduce motion, and Di-4-ANEPPS (100 μM; Thermo Fisher Scientific) was perfused to optically record membrane potentials (30). Hearts were uniformly illuminated with green excitation lasers (532 nm) to excite Di-4-ANEPPS. Emitted fluorescence was captured through a 580-nm pass filter using a complementary metal-oxide-semiconductor (CMOS) camera (MICAM Ultima, SciMedia). Movies were acquired at 1000 frames per second for a duration of 4–5 sec, with 100 × 100–pixel resolution (0.095 mm per pixel). Susceptibility to pacing-induced ventricular arrhythmia was assessed by 3 attempts of burst pacing at the apex at twice the excitation threshold (Pulsar 6i, FHM) of the left ventricle (20 Hz, amplitude 0.5–2.0 mA, 5 ms). To convert the F1759A mice to sinus rhythm, the arrhythmias were terminated by infusion of a hyperkalemia solution. After conversion to sinus rhythm, a normokalemic solution was perfused, and the optical maps were obtained.

Recorded optical movies were processed using custom software based on PV-WAVE (Precision Visuals — Workstation Analysis and Visualization Environment, Visual Numerics Inc.) (64, 65). The background fluorescence was subtracted from each frame, and spatial (5 × 5 pixels) and temporal (9 frames) conical convolution filters were used to increase signal-to-noise ratio. Movies recorded during pacing were averaged to improve signal-to-noise ratio. The optical APD were measured in each pixel at 50% repolarization level at 10Hz pacing. Phase movies and phase singularity locations were obtained after Hilbert transformation of the fluorescent signal (66). Rotational activity of at least 1 cycle was classified as a rotor (64).

### Isothermal titration calorimetry.

Experiments were performed with an ITC-200 (Microcal) at 20°C as described (11). Solution containing Na_V_1.5 C-terminal domain (20–51 μM) were titrated with 20–30 10 μL injections of solution containing FGF13 (240–510 μM). ITC experiments were repeated with different preparations and different concentrations at least 3 times to confirm thermodynamic parameters and stoichiometry values. The binding isotherms were analyzed with a single-site binding model using the Microcal Origin version 7.0 software package (Originlab Corporation). Results are presented as mean ± SEM.

### Statistics.

Results are presented as mean ± SEM. Statistical analyses were performed using Prism 8 (GraphPad Software). Data were tested using D’Agostino-Pearson normality test. For nonnormally distributed data requiring multiple comparisons, a Kruskal-Wallis test followed by a Dunn’s post hoc test were performed. For normally distributed data that required multiple comparisons testing, a 1-way ANOVA followed by a Dunnett’s test were performed. For comparisons between 2 groups, 2-tailed Student’s *t* test was used for normally distributed data, and a Mann-Whitney *U* test was used for nonnormally distributed data. Differences were considered statistically significant at values of *P* < 0.05.

### Study approvals.

The IACUC at Columbia University approved all animal experiments.

## Author contributions

JA, GSP, MBJ, and SOM designed the study. JA, DR, NC, ANK, RW, LY, BC, SIZ, JAH, UMRA, JD, CW, EYW, and MBJ performed experiments and collected the data. JA, DR, NC, ANK, RW, LY, BC, SIZ, JAH, UMRA, JD, CW, EYW, GSP, MBJ, and SOM analyzed the data. JA, DR, GSP, MBJ, and SOM wrote the manuscript.

## Supplementary Material

Supplemental data

## Version 1 09/01/2020

In-Press Preview

## Version 2 10/02/2020

Electronic publication
