# Supplementary material for: Fibroblast growth factor homologous factors tune arrhythmogenic late NaV1.5 current in calmodulin binding–deficient channels
Source: JCI Insight. 2020 Oct 2;5(19):e141736. doi: 10.1172/jci.insight.141736 (PMC7566708; doi:10.1172/jci.insight.141736)
Supplement: Supplemental data [file jciinsight-5-141736-s229.pdf]

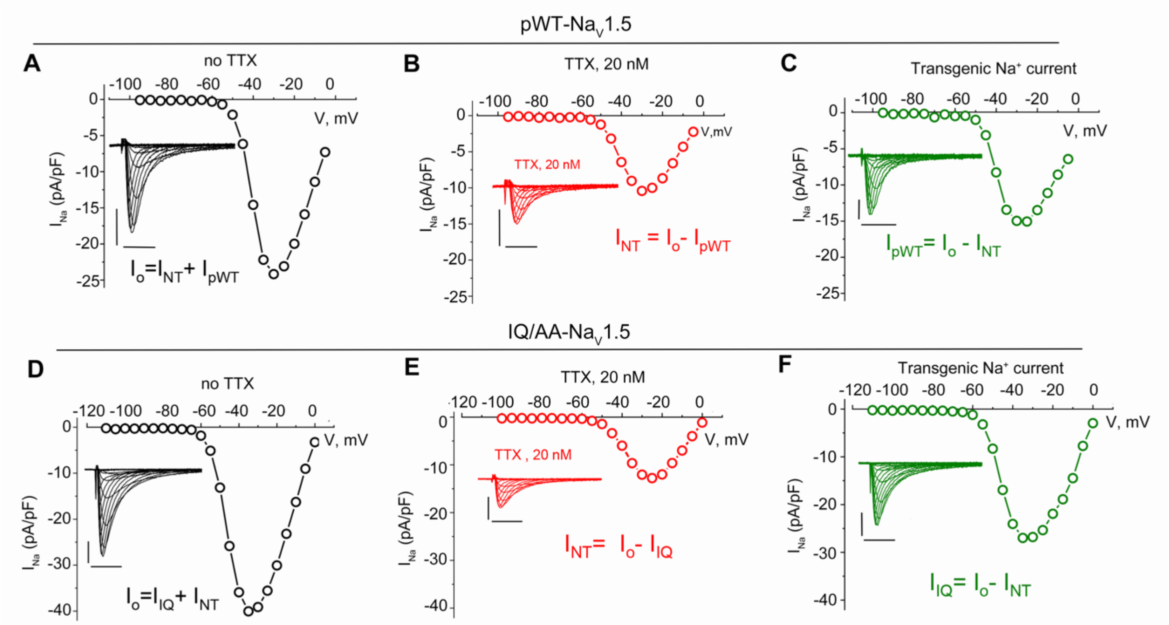

Supplemental Data Figure 1. Methodology used for analysis of transgenic Na^+ current. A,D, Graphs of current-voltage relationship for pWT Na_V1.5 and IQ/AA Na_V1.5 transgenic mice cardiomyocytes. Whole cell current traces were recorded with 3 mM Na^+ in both extracellular and intracellular solutions, in the absence of TTX. Inset, current traces from holding potential of –110 to 0 mV. Horizontal scale bar: 5 ms; vertical scale bar: 10 pA/pF. Total Na^+ current = non-transgenic (NT) current + current from pWT transgenic channels. **B,E,** Graphs of current-voltage relationship for pWT Na_V1.5 and IQ/AA Na_V1.5 transgenic mice cardiomyocytes after 20 nM TTX. The Na^+ current is from the non-transgenic (NT), endogenous channels. **C,F,** Current-voltage relationship of the pWT or IQ/AA channels, derived from total current (I_o) by subtraction of remaining current in the presence of 20 nM TTX. Representative of 21 pWT cells and 44 IQ/AA cells.

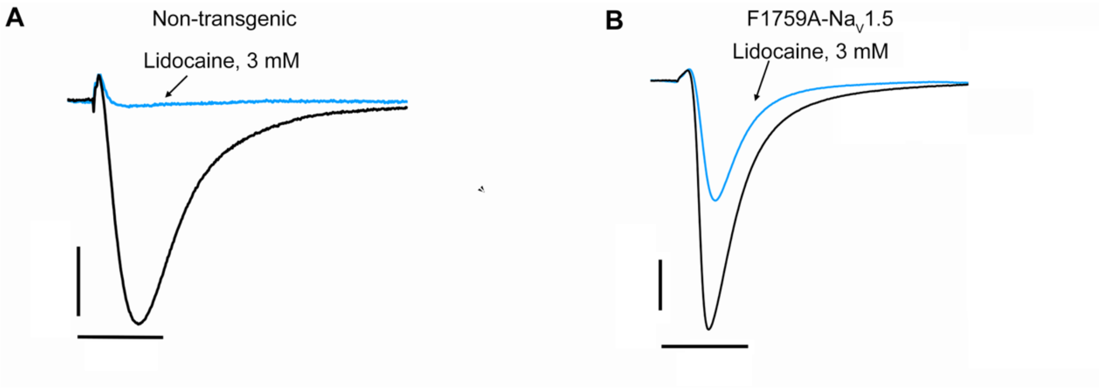

Supplemental Data Figure 2. Peak Na^+ current in F1759A transgenic mice. A-B, Exemplar whole cell Na^+ current traces of ventricular cardiomyocytes isolated from non-transgenic (A) and F1759A transgenic (B) before (black) and after 3 mM lidocaine (blue). Whole cell current traces were recorded with 5 mM Na^+ in both extracellular and intracellular solutions. Horizontal scale bars = 5 ms; vertical scale bars = 10 pA/pF. Representative of 5 similar experiments.

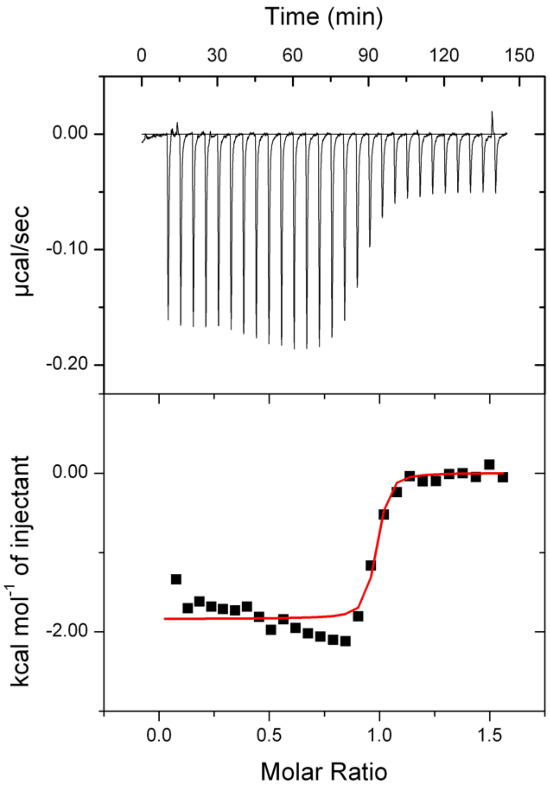

Supplemental Data Figure 3. Isothermal titration calorimetry experiment of FGF13 binding to Na_V1.5 C-terminal domain. The total heat exchanged during each injection is fit to a binding isotherm with n, K_D, and ΔH° as independent parameters. K_D =16.6 $\pm$ 0.7 nM.
